# Factors influencing critical thinking in simulation-based maternal-child nursing education among undergraduate nursing students: a mixed methods study

**DOI:** 10.1186/s12912-025-03016-w

**Published:** 2025-04-07

**Authors:** Sasitara Nuampa, Ameporn Ratinthorn, Pornnapa Tangsuksan, Thiwarphorn Chalermpichai, Kornkanok Kuesakul, Rungnapa Ruchob, Janya Chanphong, Jitrapee Buranasak, Naiyana Khadking, Kultida Subsomboon, Saowaros Pangzup, Sudhathai Sirithepmontree, Puttiraporn Hungsawanus

**Affiliations:** Department of Obstetrics and Gynaecological Nursing, Faculty of Nursing, Mahidol University, Bangkok, 10700 Thailand

**Keywords:** Critical thinking, Mixed methods design, Maternal-child nursing, Simulation, Student

## Abstract

**Background:**

Critical thinking constitutes a multifaceted and dynamic process to make appropriate decisions and solve problems. In simulation-based learning, critical thinking can be influenced by personal factors, facilitators, and design.

**Objective:**

This study was conducted to investigate the experiences and factors associated with critical thinking in simulation-based maternal-child nursing education.

**Methods:**

This study utilized an explanatory sequential mixed-methods approach. In the quantitative phase, convenience sampling was employed to select 400 undergraduate nursing students who met the following inclusion criteria: no history of repeating the maternal-child nursing and midwifery practicum course and class attendance of at least 80% of the total instructional hours. Following the completion of quantitative data collection, purposive sampling was used to recruit 80 students who had participated in and completed the initial survey to participate in focus group discussions.

**Results:**

Half of the nursing students had moderate scores of critical thinking on maternal-child nursing simulation. The regression analysis revealed that perception of professional identity, a personal factor, was statistically significantly associated with a high level of critical thinking (Beta = 0.207, t = 4.607, *p* = 0.000). Additionally, the attitude toward simulation (Beta = 0.139, t = 2.731, *p* = 0.007) and perceived stress (Beta = -0.103, t = -2.269, *p* = 0.024) were statistically significantly associated with critical thinking level. In the simulation design, the support aspect toward simulation design (Beta = 0.265, t = 2.943, *p* = 0.003) and the problem-solving aspect toward simulation importance (Beta = 0.239, t = 2.288, *p* = 0.023) were statistically significantly associated with a high level of critical thinking. The multiple linear regression model accounted for 35% of the variance in critical thinking with maternal-child nursing simulated learning. Qualitative data revealed the following themes: (1) a well-planned approach enables me to optimize my learning; (2) allow me to make mistakes, but please don’t leave me with failure; and (3) emulating practice shapes my growth as a nursing professional.

**Conclusions:**

The personal factor and simulation design factors were important for increasing critical thinking level. Promoting effective learning strategies, such as the use of simulated experiences, is useful in enhancing midwifery and nursing students’ competencies.

**Supplementary Information:**

The online version contains supplementary material available at 10.1186/s12912-025-03016-w.

## Introduction

Simulation-based learning (SBL) is an educational approach that enables learners to encounter real patient scenarios without subjecting actual patients to the inherent risks associated with student learning [1]. According to the present health education landscape, there’s a rising demand for clinical placements alongside a scarcity of available practice supervisors. Concurrently, ethical concerns regarding patient care and safety persist, including the imperative for enhanced healthcare quality at reduced costs and shorter hospital stays. Consequently, students face diminished opportunities for active engagement in patient care and handling practice scenarios [2–4]. Simulation methods afford learners the chance to engage in experiential learning within a secure environment [5], facilitating the exploration of patients’ issues and the development of clinical reasoning and judgment skills through practice [6]. To create these skills, including the ability to work as a team, self-assessment, and using critical thinking while dealing with actual, real patients among nursing students, many institutions have used SBL in the nursing curriculum [7]. A wide range of low- to high-fidelity simulation methods are available in nursing education, and their effectiveness is confirmed [8].

Employing human-like mannequins in simulation proves to be an effective strategy for enhancing the critical thinking and clinical decision-making abilities of novel nurses [9]. According to the previous study, patient simulators could improve the nursing students’ critical thinking skills and help them implement theory in clinical practice [10]. Moreover, several studies found that simulation had positively affected the critical thinking skills among nursing students [11–16]. A wide range of simulation methods were examined, such as electronic interactive simulation programs [12], video simulation [17], and high-fidelity and low-fidelity programs [16].

Critical thinking constitutes a multifaceted and dynamic process shaped by attitudes and strategic skills, all oriented towards the attainment of particular goals or objectives [18]. In the context of health sciences and clinical environments, the development of critical thinking has been associated with concepts such as critical reasoning [19], clinical judgment [20], and reflective practice [21]. Both clinical reasoning, signifying the process, and clinical judgment, which denotes the diagnosis derived from this process, are integral components of critical thinking within clinical settings [22]. Nurses and nursing students as health care providers should be creative, self-directed, and critical thinkers to be able to make appropriate decisions and solve clinical problems they are encounter [4]. Given the importance of critical thinking in nursing, nurse educators should use teaching methods that can foster this ability in nursing students [23].

Recent studies have explored essential factors related to student critical thinking during learning with simulation according to the National League for Nursing/Laerdal Jeffries Simulation Theory, which is composed of educational practices, facilitators, participants, simulation design characteristics, and expected outcomes [24]. The framework is useful for developing, implementing, and evaluating simulation-based activities in midwifery and nursing education. Factors that affect learning critical thinking in SBL included personal factors such as previous learning outcomes [25], attitude toward SBL [26, 27], professional identity [28], perceived stress [29], and facilitator factors; teaching competencies [30]; and simulation design factors [31].

Attitude toward SBL reflects students’ perceptions and engagement with simulation as an educational method, influencing their motivation and learning outcomes [26, 27]. Professional identity shapes students’ self-perception and integration into the nursing profession through education and clinical experiences [28]. Perceived stress refers to students’ subjective evaluation of stressful situations, affecting their confidence and ability to engage in critical thinking during simulation. Facilitator factors, including teaching competencies, contribute to effective learning by providing structured guidance and psychological safety [30]. Additionally, simulation design factors include objectives, fidelity, problem-solving, learner support, and debriefing, which collectively influence students’ learning effectiveness and ability to apply critical thinking in clinical scenarios [31]. These factors enhance critical thinking skills in SBL.

During the COVID-19 pandemic, midwifery and nursing education faced disruptions, especially in clinical practices, which were found to have a significant impact on students’ confidence in their academic success [32]. Responding to this crisis, the midwifery and nursing school in this study endeavored to adopt simulation-based learning despite resource limitations, aiming to support midwifery and nursing students and enhance their proficiency in maternal and child care. This study aimed to investigate factors associated with critical thinking in SBL among midwifery and nursing students and explore their learning experiences with critical thinking skills.

## Materials and methods

### Study design

**Fig. 1:**
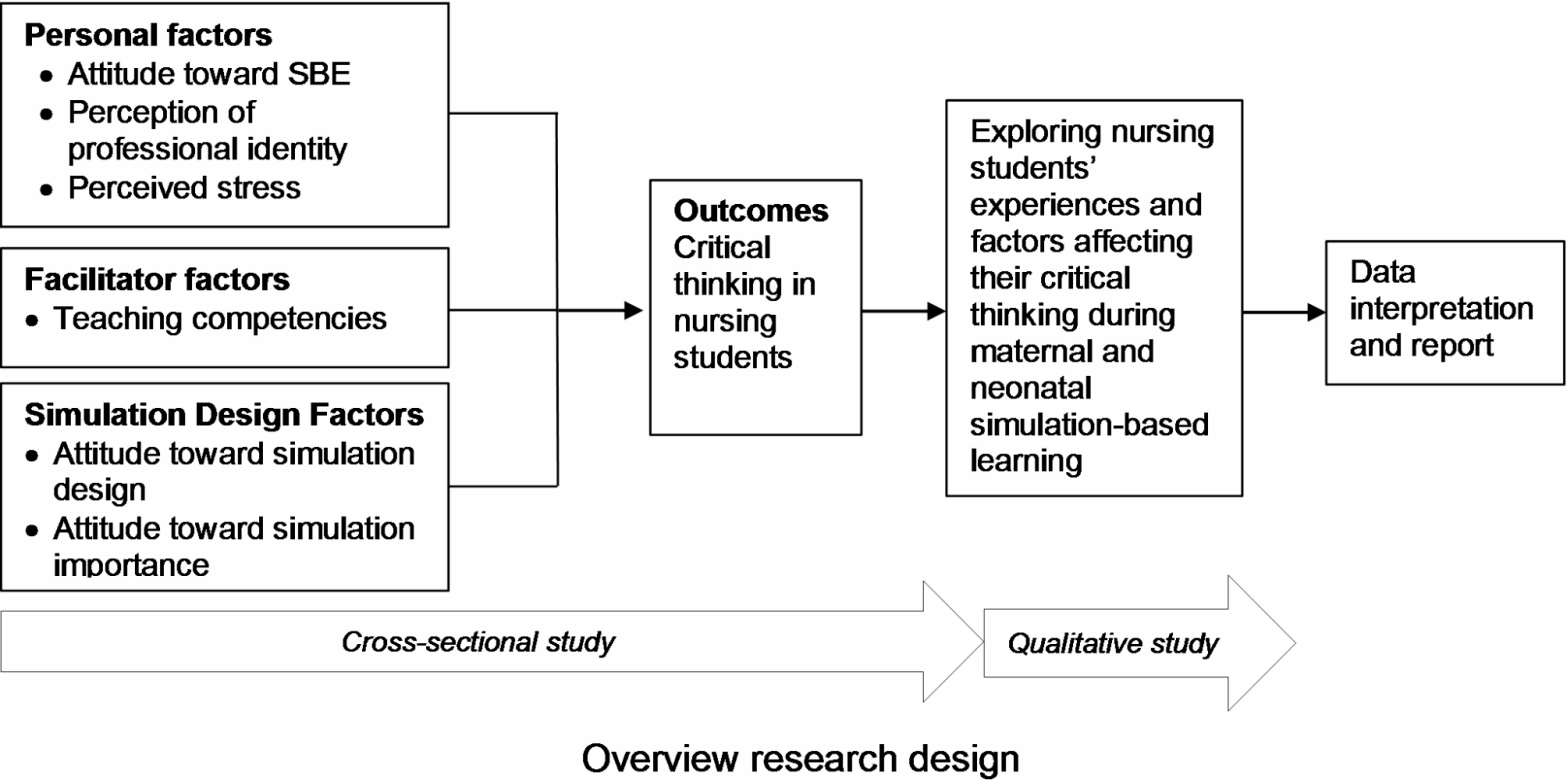
Overview research design. SBE, Simulation-based education

This study was part of a major study, “The Study of Practicing Learning Outcomes from Clinical Simulation in Maternity-Newborn Nursing and Midwifery Practicum among Nursing Students.” The previous publication reported antenatal simulation-based learning on satisfaction and self-confidence levels [33]. The mixed-methods design followed an explanatory sequential approach [34]. Initially, a cross-sectional survey was administered to evaluate the factors influencing nursing students’ critical thinking levels in the SBL of maternal and neonatal practice. This was followed by a qualitative study in which focus group interviews were conducted to explore the experiences and factors that affected the critical thinking skills of those who completed maternal and neonatal SBL. The quantitative and qualitative results were interpreted and reported through narrative outcomes (Fig. 1).

### Setting and participants

The sample size was calculated using the G*power 3.1.9.4 software with the following command for linear multiple regression: effect size f^2^ = 0.07 [23], an alpha level of 0.05, a power of 0.95, and 14 predictors. The calculated sample size is 400 cases. The convenience sampling was employed to select 400 undergraduate nursing students from the third-year students (*n* = 200) and fourth-year students (*n* = 200) who completed the courses Maternity-Newborn Nursing and Midwifery Practice I and II, respectively. Inclusion criteria included (a) full-time Thai national students, (b) not previously enrolled in the Maternity-Newborn Nursing and Midwifery Practice course, and (c) having a smartphone or other electronic device to complete the online questionnaire. After completing the online survey, purposive sampling was used to recruit those who had participated in and completed the initial survey to participate in focus group discussions.

This study was conducted at an urban university in Bangkok, involving students enrolled in a baccalaureate nursing program. The maternal and neonatal simulation-based learning is the practical part of the Maternity-Newborn Nursing and Midwifery Practice course. In the course of Maternity-Newborn Nursing and Midwifery Practice, I served 3 credits aimed at promoting the competencies of antenatal care and postpartum nursing care. Moreover, Maternity-Newborn Nursing and Midwifery Practice II had 4 credits with the subject objective of promoting the competencies of intrapartum care and maternal complications care in nursing students. All simulation scenarios were designed and validated by the instructor team according to the course learning outcomes. The onsite simulation was held at the Learning Resource Center. Firstly, antenatal practical skills for nursing students consisted of an antenatal physical and mental assessment, an abdominal examination for pregnant women over 28 weeks of gestation, and counseling for promoting healthy pregnancy and managing common discomforts. Secondly, intrapartum practical skills included conducting the normal delivery, neonatal care and management, and obstetric emergency nursing management. Thirdly, postpartum nursing care consisted of maternal and child assessment, maternal education, and breastfeeding. Finally, obstetric complications set the nursing management in emergency situations of pre-eclampsia and eclampsia, abortion nursing care, and mental illness care. The simulation-based learning that was designed consists of three stages: (a) pre-brief/introduction; (b) simulation running; and (c) debriefing [35].

### Data collection

Data were collected between May and December 2022. This process was conducted without coercion after finishing the Maternity-Newborn Nursing and Midwifery Practice course via an online cross-sectional survey. After the announcement of course results, the research assistant distributed an infographic to inform the project and recruit volunteers via the students’ LINE application group. LINE, a widely used messaging platform in Thailand, was utilized to efficiently distribute study information and survey links, facilitating seamless participant recruitment while ensuring convenience and autonomy in participation. A survey Google Form link was provided so that participants can freely decide whether they wish to participate and continue answering the online questionnaires. Participants who were willing to participate in this study were recruited and signed online consent forms. The online questionnaire was asked to be completed within 48 h. After completing the questionnaire, participants received a souvenir worth 50 baht (approximately 2 US dollars). Volunteer participants who completed the online survey were invited to participate in 45–60-minute virtual focus group discussions (FGDs) via Microsoft Teams. The platform’s features, including real-time discussions, screen sharing, and secure data management, facilitated efficient qualitative data collection. Eighty students were separated into sixteen FGDs (five students per group) depending on their satisfaction levels. There were eight groups of low-to-high satisfaction scores. Four settings of antenatal care, intrapartum care, postpartum care, and complications were focused on simulation learning experiences with twenty participants per situation. Before the discussion, the researcher obtained permission for audio recording, followed by verbatim transcription and concurrent data analysis to ensure research rigor. Data saturation was ensured through an iterative process of data collection and analysis, whereby interviews continued until no new themes or insights emerged. Field notes and a codebook were used for the analysis.

### Ethical approval

The study was conducted in accordance with the Declaration of Helsinki and approved by the Institutional Review Boards (IRB or Ethics Committees). The online informed consent was obtained from all participants who agreed to participate in the study. Moreover, their responses to the questionnaire did not affect their final scores in the course. All data were collected anonymously.

### Measures

A self-administered online survey, using standardized methods and consisting of three sections, was sent to the students via three different links. A total of seven questionnaires were administered, which were received with permission from original studies as shown in Table 1. This study established content validity through expert review by three nursing education specialists (CVI = 0.80–1). Following IRB approval, reliability testing with 20 comparable participants confirmed high internal consistency, with Cronbach’s alpha exceeding 0.80 for all seven questionnaires. We used parts of these questionnaires to measure factors that predict nursing students’ satisfaction and self-confidence in the antenatal simulation [33]. Moreover, a 15-question semi-structured focus group guide was used to collect students’ experiences on the simulations in the Maternity-Newborn Nursing and Midwifery Practice course, which included the following questions: How did you feel after the practice simulation? What were your expectations or goals? How did you achieve them? (Supplementary File 1). Two nursing education experts and one qualitative expert reviewed the instruments for content validity. Based on their evaluation of content accuracy and linguistic appropriateness, the instruments were refined and revised accordingly.

**Table 1:** List of self-administered online questionnaires

Measurement	Specific measure	Cronbach’s alpha, this study
Demographic questionnaire	Age, Gender, Cumulative GPA, Maternity-Newborn Nursing and Midwifery lecture grade, SBE experience	N/A
Attitude scale toward simulation-based education	*18* items, 5-point Likert scales ranging from 1 (strongly disagree) to 5 (strongly agree), range 18–90, a higher score indicating a high level of attitude toward simulation-based education. The Cronbach’s alpha coefficient test resulted in a value of 0.72 [27].	0.876
Professional identity scale for nursing students	*1*7 items, 5-point Likert scales ranging from 1 (strongly disagree) to 5 (strongly agree), range 17–85, a higher score indicating a high level of positive perception of professional identity. The Cronbach’s alpha coefficient test resulted in a value of 0.83 [36].	0.873
Perceived stress scale	*1*0 items, 5-point Likert scales ranging from 0 (almost never) to 5 (almost always), range 0–50. 1–13 scores indicate a low level of stress, 14–26 indicate a mild level of stress, and 27–40 scores indicate a high level of stress. Demonstrated reliability and validity [37, 38]. The Cronbach’s alpha coefficient test resulted in a value of 0.87 [37].	0.802
Evaluation of teaching competencies scale	9 items, 3-point Likert scales ranging from 1 (disagree) to 3 (agree), range 9–27, a higher score indicating a high level of positive perception of teaching competencies. The Cronbach’s alpha coefficient test resulted in a value of 0.94 [39].	0.900
Simulation design scale: student version	Two parts of the questionnaires were attitude toward simulation design (19 items) and perceived important design (19 items), 5-point Likert scales ranging from 1 (strongly disagree) to 5 (strongly agree), range of 19–95 for each part, a higher score indicating participants perceived the design of simulation with a high degree of suitability and importance. The Cronbach’s alpha coefficient test for the perception of SBE design was 0.92, while the perception of importance was 0.96 [40].	0.969/0.979
Critical thinking (Yoon’s Critical Thinking Disposition Instrument)	27 items, 5-point Likert scales ranging from 1 (strongly disagree) to 5 (strongly agree), range 27–135, a higher score indicating a high level of critical thinking. The Cronbach’s alpha coefficient test resulted in a value of 0.84 [23].	0.906

GPA, grade point average; SBE, simulation-based education; N/A, Not Applicable

### Data analysis

The data were analyzed using SPSS version 20. Descriptive statistics, including the mean and standard deviation (SD), were used for variables that met the assumption of normality, as assessed by the Skewness-Kurtosis test. Linear correlation, assessing the relationship between two variables measured on an interval or ratio scale, was determined using Pearson’s correlation coefficient (r). Multiple linear regression analyses were conducted to account for potential influences on students’ critical thinking in maternal and neonatal care during simulation-based learning. Various factors, including personal factors (attitude toward SBE, professional identity, and perceived stress), facilitator factors (teaching competencies), and simulation design factors (attitude toward simulation design and perceived importance of design), were examined as independent variables in the regression analysis. Statistical significance was set at a level of 0.05. The analysis met all multiple regression assumptions. Linearity was confirmed via scatterplots, independence of errors by a Durbin-Watson statistic (1.5–2.5), and homoscedasticity through a residual scatterplot. Multicollinearity was ruled out, with Pearson correlations (0.25–0.53), VIF < 10, and tolerance > 0.1, supporting model validity.

Qualitative analysis involved conducting manual content analysis on verbatim transcriptions of recorded interviews, each averaging 45–60 min. Following the interviews, field notes were taken to capture initial impressions. The analysis was carried out collaboratively by five members of the research team (SN, AR, JB, TC, and PT), each offering diverse perspectives on simulation-based learning. Individual analysis of transcripts was undertaken, involving a line-by-line reading and coding process to identify key concepts. These smaller codes were then grouped into larger categories, which were further organized into major themes. A concurrent data collection and analysis approach was employed to delve into new concepts emerging from subsequent interviews in detail [41].

### Quantitative results

#### Demographic and variable data in the quantitative results

The study’s findings revealed that the average age of participants was 22 years (SD = 1.11), with a range of 19–28 years old. The majority of students were female (*n* = 360, 90%), while 10% (*n* = 40) were male. The grade point average (GPA) had an average of 3.11 points (SD = 0.34) and a range of 2.24–3.90 points. Table 2 presents the independent and dependent variables as percentages and separates them into dimensions and categories. The cut-off values of perceived stress and critical thinking level were used at the 25th and 75th percentiles. Especially, the critical thinking levels in this study found that half of the students had a moderate level of critical thinking in midwifery SBL. Nearly 27% had a low level of critical thinking, while 23.50% (*n* = 94) presented a high level of critical thinking.

**Table 2:** Correlation of personal factors, facilitator factors, simulation design factors, and critical thinking outcome (*n* = 400) with critical thinking

Factors	Range	Min-max	Mean (SD)	*N* (%)	*r*	*P*-value
***Personal Factors***						
**Attitude toward Simulation-based education**	18–90	40–90	72.03 (9.83)		0.369	0.000
Satisfaction– self-confidence	6–30	12–30	24.63 (3.41)		0.354	0.000
Clinical competency– self-efficacy	5–25	9–25	20.46 (3.15)		0.372	0.000
Seriousness - fidelity	4–20	7–20	15.81 (2.63)		0.341	0.000
Barriers– difficulties	3–15	3–15	11.13 (2.38)		0.153	0.002
**Perception of professional identity**	17–85	27–79	55.49 (8.83)		0.373	0.000
**Perceived stress**	0–40	0–35	16.12 (5.35)		-0.300	0.000
Mild level	0–13			122 (30.50)		
Moderate level	14–26			266 (66.50)		
Severe level	27–40			12 (3.00)		
***Facilitator Factors***						
**Perceived teaching competencies**	9–27	11–27	24.08 (3.38)		0.273	0.000
Communication	1–3	1–3	2.70 (0.49)		0.199	0.000
Availability	1–3	1–3	2.75 (0.47)		0.137	0.006
Creativity	1–3	1–3	2.72 (0.49)		0.213	0.000
Individual consideration	1–3	1–3	2.63 (0.52)		0.190	0.000
Social awareness	1–3	1–3	2.63 (0.52)		0.198	0.000
Feedback	1–3	1–3	2.57 (0.54)		0.141	0.005
Professionalism	1–3	1–3	2.73 (0.47)		0.177	0.000
Conscientiousness	1–3	1–3	2.70 (0.49)		0.170	0.000
Problem-solving	1–3	1–3	2.65 (0.52)		0.161	0.001
***Simulation Design Factors***						
**Attitude toward simulation design**	19–95	22–95	74.99 (11.49)		0.497	0.000
Objectives/information	5–25	6–25	19.81 (3.24)		0.478	0.000
Support	4–20	4–20	16.29 (2.65)		0.490	0.000
Problem solving	5–25	7–25	19.95 (3.28)		0.463	0.000
Feedback	4–20	4–20	16.10 (2.58)		0.432	0.000
Fidelity	1–5	1–5	3.88 (0.82)		0.340	0.000
**Attitude toward simulation importance**	19–95	17–95	75.73 (13.06)		0.376	0.000
Objectives/information	5–25	5–25	20.51 (3.28)		0.325	0.000
Support	4–20	4–20	16.33 (2.82)		0.338	0.000
Problem solving	5–25	5–25	20.41 (3.46)		0.342	0.000
Feedback	4–20	5–20	16.38 (2.74)		0.316	0.000
Fidelity	1–5	0–5	4.05 (0.82)		0.253	0.000
***Outcome***						
**Critical thinking level**	27–135	74–131	100.87 (11.65)			
Low level	27–92			106 (26.50)		
Moderate level	93–108			200 (50.00)		
High level	109–135			94 (23.50)		
**Critical thinking disposition**						
Confidence	4–20	7–20	14.12 (2.32)			
Eager	5–25	9–25	19.30 (3.06)			
Fairness	4–20	4–20	16.35 (2.44)			
Objectivity	3–15	7–15	12.14 (1.89)			
Prudence	4–20	7–20	13.58 (2.43)			
Scepticism	4–20	7–20	13.73 (2.42)			
Systematicity	3–15	6–15	11.52 (1.90)			

p-value < 0.05

#### Correlation for critical thinking on simulation-based learning in maternal and neonatal nursing practice

The correlation analysis reveals a statistically significant relationship between personal, facilitator, and simulation design factors and students’ critical thinking in midwifery SBL. Notably, simulation design factors, including attitude toward midwifery simulation-based education (*r* = 0.497, *p* = 0.000) and attitude toward simulation importance (*r* = 0.376, *p* = 0.000), exhibited moderately positive correlations with students’ critical thinking. Within the realm of attitude toward simulation design, dimensions such as support (*r* = 0.490, *p* = 0.000) and problem-solving (*r* = 0.342, *p* = 0.000) showed statistically significant correlations with students’ critical thinking. Moreover, perceptions of professional identity (*r* = 0.373, *p* = 0.000) and attitude toward simulation-based education (*r* = 0.369, *p* = 0.000) displayed weak positive correlations with students’ critical thinking, with clinical competency and self-efficacy demonstrating the strongest correlation (*r* = 0.372, *p* = 0.000) among attitude factors. Conversely, perceived stress exhibited a weak negative correlation with students’ critical thinking (*r* = -0.300, *p* = 0.000), indicating a disadvantageous impact. Finally, teaching competencies, as a facilitator factor, displayed a weak positive correlation with students’ critical thinking (*r* = 0.273, *p* = 0.000), as illustrated in Table 2.

#### Predictive factors for students’ critical thinking with simulation-based learning in midwifery practice

Table 3 shows personal, facilitator, and simulation design factors associated with students’ critical thinking with simulated learning in midwifery practice. The regression analysis revealed that perception of professional identity, a personal factor, was significantly associated with a high level of critical thinking (Beta = 0.207, t = 4.607, *p* = 0.000). Additionally, attitude toward simulation-based education (Beta = 0.139, t = 2.731, *p* = 0.007) and perceived stress (Beta = -0.103, t = -2.269, *p* = 0.024) were significantly associated with critical thinking level. In simulation design, the support aspect of attitude toward simulation design (Beta = 0.265, t = 2.943, *p* = 0.003) and the problem-solving aspect of attitude toward simulation importance (Beta = 0.239, t = 2.288, *p* = 0.023) were significant associated with a high level of critical thinking. The multiple linear regression model accounted for 35% of the variance in critical thinking with midwifery simulation learning (adjusted R^2^ = 32.0%).

**Table 3:** Factors predicting students’ critical thinking in maternal and neonatal practical skills (*n* = 400)

Factors	B	SE	Beta	T	*p*	95% CI
Attitude toward simulation-based education	0.165	0.060	0.139	2.731	0.007*	0.046, 0.284
Perception of professional identity	0.274	0.059	0.207	4.607	0.000*	0.157, 0.390
Perceived stress	-0.224	0.099	-0.103	-2.269	0.024*	-0.418, -0.030
Perceived teaching competencies	-0.318	0.187	-0.092	-1.700	0.090	-0.685, 0.050
Attitude toward simulation design:						
objectives/information	0.399	0.339	0.111	1.179	0.239	-0.267, 1.066
support	1.164	0.396	0.265	2.943	0.003*	0.386, 1.943
problem solving	0.062	0.349	0.018	0.178	0.859	-0.625, 0.749
feedback	0.211	0.386	0.047	0.546	0.585	-0.548, 0.970
fidelity	-0.512	0.935	-0.036	-0.547	0.585	-2.350, 1.327
Attitude toward simulation importance:						
objectives/information	-0.559	0.390	-0.157	-1.433	0.153	-1.327, 0.208
support	0.260	0.465	0.063	0.559	0.576	-0.654, 1.175
problem solving	0.799	0.349	0.239	2.288	0.023*	0.113, 1.486
feedback	-0.420	0.474	-0.099	-0.886	0.376	-1.353, 0.513
fidelity	-0.522	0.996	-0.037	-0.524	0.600	-2.480, 1.436

*R* = 0.59; R^2^ = 0.35; adjusted R^2^ = 0.32

B = beta coefficient; CI = confidence interval; SE = standard error; t = t-score of a regression model

*p value < 0.05

### Qualitative results

The qualitative content analysis revealed that midwifery and nursing students provided their learning experiences related to critical thinking skills in SBL, while promoting factors and barriers were disclosed. Students described three themes according to simulation design, personal aspects, and facilitators on critical thinking skills. Three themes consisted of: a well-planned approach enables me to optimize my learning; allow me to make mistakes, but please don’t leave me with failure; and emulating practice shapes my growth as a professional.

#### A well-planned approach enables me to optimize my learning

The simulation design should be comprehensive, including the preparation stage, learning stage, and assessment. In this study, students shared their experiences and emphasized the significance of adequate preparation for simulation learning and the importance of thoughtful simulation design. Initially, they highlighted the necessity of understanding and readiness before engaging in practice. Furthermore, they underscored the need for realistic and varied SBL experiences, with scenarios progressing from basic to complex levels, and emphasized the importance of allocating sufficient time during the learning process. They described the vital influence of simulation design on their critical thinking skills, such as systematic thinking, reasonableness, analyzing and finding solutions, and increasing their confidence for clinical practice.

Several students mentioned they knew what they had to learn in simulation class. They were able to prepare their specific knowledge, which reduced their nervousness and stress and increased their confidence to solve the situation.

*“Before we start the training*,* the teacher kind of gives us an idea*,* like saying*,* ‘What are we going to do tomorrow or something like that?’ It’ s like he gives us a heads-up so that we can review beforehand. So*,* when we arrive*,* we won’t…not be standing there confused or anything like that.” (group 13*,* complication SBL)*

Regarding the learning stage, several students described their experiences and needs about midwifery simulation design in terms of creating realistic simulation scenarios, diverse situations, grading scenarios, and providing enough time. Moreover, they suggested that scenarios should include soft skills as part of working with a team and provide sufficient time for self-directed learning and preparation.

*“I think if we’re not confident in handling common*,* easy cases we frequently encounter on the ward*,* encountering difficult cases… well*,* even dealing with ordinary matters*,* we’re still unsure. So*,* how can we be confident in handling difficult situations? I think if we practice general topics thoroughly and accurately*,* first and then gradually move on to increasingly difficult cases*,* step by step*,* starting with real-life scenarios*,* and mastering them first*,* before moving on to more complex issues*,* I will be confident that I can handle them without feeling like I’ve failed.” (group 6*,* postpartum SBL)*

*“The group learning process works well in that it doesn’t rely solely on individual thinking. We also get to see our peers because we have to provide feedback to them. We observe our peers as examples*,* so by looking at our peers*,* we train ourselves to think analytically multiple times.” (group 4*,* antenatal SBL)*

Finally, some students suggested that the assessment stage should mention student competencies, not count the number of practices. The students needed the freedom to self-judge their readiness for an assessment.

*“In class*,* the teacher might say how many times we have to take this exam*,* how many times for that one. Instead of enforcing it like this*,* I think it adds pressure. It would be better to give us the opportunity to decide when we’re ready and then take the exam once. It’s about reaching a mutual agreement beforehand. If I can do it*,* then I pass.” (group 9*,* intrapartum SBL)*

#### Allow me to make mistake, but please don’t leave me with failure

Students in this study shared their feelings and experiences regarding the strengths of the simulation experience, which allowed them to make mistakes and repeat actions until they gained confidence. However, some students faced obstacles hindering their ability to learn from mistakes and failure. Fortunately, the benefits of debriefing sessions were found to support the enhancement of their confidence and critical thinking during simulation.

Simulation-based learning might induce pressure, and some students expect high competencies, which can be stressful. Some students accepted that stress could prevent the learning process and critical thinking outcomes. Many students mentioned that facilitator characters should create a positive environment and offer a chance during simulation.

*“I feel that simulation scenarios add pressure to myself. When we participate*,* it’s like we’re under pressure*,* and we don’t dare to answer or do anything*,* even though sometimes we know the answer. I think the issue of solving this problem lies within the student themselves and also with the teachers*,* in how they can talk to us to make us feel confident and give us the opportunity to make mistakes.” (group 8*,* postpartum SBL)*

*“…It’s like when one group encounters difficulties and the teacher ends it*,* then assigns another group to replace them. I feel like it may be cutting off the learning opportunities for all students*,* potentially undermining the confidence of the first group. It’s similar to coming to learn for the first time*,* teacher. Perhaps you should give us a chance to give us a hint*,* just a little*,* so we can learn together.” (group 15*,* complication SBL)*

Some students expressed that they need positive reinforcement from teachers or facilitators, which could increase their confidence to learn and dare to make decisions.

*“What helps to achieve success is*,* I believe*,* the praise from the teacher*,* which greatly influences learning. Some people might feel demoralized if they receive negative feedback after making a mistake once*,* and they might not dare to do it again. But if the teacher praises us*,* we feel more motivated and courageous to change*,* to act*,* and to think.” (group 4*,* antenatal SBL)*

In addition, debriefing serves as an encourage process and facilitates students achieving critical thinking skills. Almost all students in this study described the important influence of instructors who were facilitators in scenario practices. They preferred a facilitator who provided a suitable reason, systematic summary, and linkage with theory to ensure applicable skills, and friendly support.

*“The atmosphere is also a factor in our effective learning. If we can ask or discuss directly with the teacher*,* that helps a lot. I’m someone who remembers things better if I ask or explain right there; it’s much better than going back to find it myself. Also*,* we don’t know if what we find is correct or not.” (group 11*,* postpartum SBL)*

*“The way the teachers use language when explaining is as if we have the right to make mistakes. They speak to us in a tone that isn’t pressuring or displeased with what we can’t do. They gradually explain step by step what needs to be done and then provide reasons for doing so. I think this is a good thing because just encountering childbirth is already stressful. Even though it’s not actual childbirth*,* we still feel the pressure.” (group 10*,* intrapartum SBL)*

#### Emulating practice shapes my growth as a professional

This study found that several students perceived a positive attitude towards the midwifery simulation. They disclosed that scenario practices increased their confidence to provide nursing care in a real-life situation. They perceived applicable skills through similar practice in simulation.

*“In simulation scenarios*,* what we gain is increased knowledge and skills compared to before we trained. We know what to do next when faced with real situations. We might not have to follow those exact steps; instead*,* we can just control the situation to ensure the safety of both the mother and the baby. It’s like we truly know more.” (group 5*,* postpartum SBL)*

*“By experiencing this*,* when I actually go to the ANC [Antenatal Care] clinic*,* I feel more confident. Then*,* I can perform well*,* with fewer mistakes than when I practiced in the classroom or perhaps there are no mistakes at all.” (group 2*,* antenatal SBL)*

Moreover, some students shared their successful experiences in the clinical setting through acceptance and trust from patients. This could bring value and a positive professional identity.

*“My success is being able to provide advice and answer questions during prenatal check-ups. This is my achievement. When we go up to the ward*,* we can talk and provide advice to the mothers*,* and they believe in us*,* as if they have confidence in nursing students. That’s my success.” (group 3*,* antenatal SBL)*

## Discussion

The findings of this study highlighted the influence of students’ personal factors and key elements of simulation design on the level of critical thinking among students engaged in midwifery practices. Furthermore, qualitative results underscore the clear preference for SBL among students, particularly when designed appropriately to foster confidence and critical thinking. Emphasizing the debriefing process and maintaining a positive learning environment are crucial considerations for facilitators to overcome obstacles to critical thinking outcomes.

Critical thinking is conceptualized as a purposeful and self-regulatory judgment [42]. For nurses, critical thinking is the cornerstone of evidence-based practice and high quality of care [43]. This study found professional identity was one of the important factors influencing critical thinking in midwifery simulated learning. Students in this study passed the clinical practices and learned the professional roles. Professional practice experience and having good models are identified as key factors for students’ professional identity development [44]. Professional identity in nursing students is a prerequisite to the development of nursing competence [45]. If nursing students feel like nurses, as a vital component of developing their clinical judgment in practice [20]. Moreover, giving nursing students a safe environment to practice in is a key factor in learning and reducing stress [46]. Simulation-based education has been an important mechanism used to provide clinical training and experience to students in a safe space and learn from error to grow skill sets and confidence with experiences [47].

Moreover, stress levels in this study affected reducing critical thinking among students during simulation learning. According to the previous study, high levels of stress inhibit nursing students’ ability to use critical thinking [43]. Educators usually rely on teacher-centered strategies, time constraints, and understaffing, which do not develop critical thinking skills [42, 43]. Stress has the potential to cause cognitive impacts, including reduced attention and concentration, memory decline, heightened error rates, and challenges in responding promptly and effectively [48]. Furthermore, attitudes toward simulation learning had a significant influence on critical thinking. Students perceived the simulation was able to improve students’ self-esteem by providing an opportunity to practice in a risk-free environment. According to Maxson et al. [49], the simulation environment enables students to surpass the constraints of clinical practice, enhances coping abilities, refines critical thinking skills, and facilitates rapid adaptation to real-world clinical settings. These benefits of SBL positively affect nursing students approaching the transition to registered nurses, which results in a high quality of competency [50].

In terms of simulation design being a critical factor, this study revealed the importance of emphasizing support aspects and problem-solving skills within the learning plan. Moreover, students perceived that preparation, a friendly learning process, and debriefing affected their ability to think critically. Simulation, as an educational method, entails the effective integration of real-world elements to attain targeted learning or assessment objectives. Hence, educators utilizing simulations in learning environments must employ pedagogical approaches that promote reciprocal transfer of learning between classroom and clinical contexts while also nurturing critical thinking skills and facilitating clinical reasoning abilities [51]. Effectively structured instructional and curriculum designs, such as problem-based learning, have been suggested as mechanisms for cultivating and evaluating various behaviors, including critical thinking skills [52]. The materials utilized should embody authenticity, mirror real-world practices, and offer clarity and guidance to students regarding time, location, and roles. They should encompass social, political, and ethical dimensions while also acknowledging the potential of multimedia to enhance the fidelity of simulations [53]. Furthermore, Song and Lim [54] examined various forms of debriefing and concluded that structured debriefing is the most suitable approach for fostering self-reflection, integrating critical thinking, and enhancing problem-solving abilities. They found that structured debriefing better equips learners to make sound decisions in clinical scenarios compared to alternative forms of debriefing, such as video or written formats. While several previous studies showed a positive effect of simulation training on critical thinking through an electronic interactive simulation program [11], high-fidelity simulation [12], and video simulation scenarios [17]. A suitable simulation design has an important impact on achieving optimal learning outcomes.

In the current nursing educational environment, characterized by rapid technological evolution and changing clinical practice patterns post-COVID-19, instructors should further explore integrating advanced simulation technologies into curricula. For instance, VR and AR-based simulations provide opportunities for repetitive, safe, and error-tolerant practice, allowing students to experience clinical scenarios not always feasible in traditional laboratory settings [55, 56]. However, despite technological advancements, instructors must remain mindful of several critical considerations. First, technological sophistication alone does not guarantee improved learning outcomes. The effectiveness of advanced simulations depends deeply on thoughtful instructional design, pedagogical alignment, learner-centred facilitation, and clear educational objectives [57]. Second, instructors should consider learners’ psychological readiness and provide sufficient support, ensuring that innovative simulation environments do not become sources of stress but rather promote learner confidence and professional identity formation, as identified in the qualitative findings of this study. Therefore, educators planning future SBL approaches must integrate advanced technologies judiciously, ensuring that innovation remains guided by pedagogical principles, learner needs, psychological support mechanisms, and clear instructional goals.

Future research should prioritize exploring key factors related to students’ personal characteristics, such as cultivating a positive attitude towards simulation learning, reducing stress levels, fostering a strong sense of professional identity, and sequencing activities to boost confidence. Additionally, there is a need to tailor the simulation design to align with students’ preferences, thereby promoting a more positive learning experience. These considerations are essential for the effective implementation of simulation-based education, especially in the context of midwifery practices for undergraduate students.

This study has several limitations. Since the data collection was based on self-administered online surveys where participants were asked about their past simulation learning experience, recall bias may exist. However, a strength of this study lies in its use of a mixed-methods research design, which may reduce self-reported bias and strengthen the analysis [58], resulting in comprehensive research outcomes and a better understanding of the learners’ experiences.

## Conclusions

Both individual and simulation design factors were important for increase critical thinking level in midwifery simulation learning among midwifery and nursing students. Promoting effective learning strategies, such as use of simulated experiences are useful in enhancing midwifery and nursing students’ competencies.

## Electronic supplementary material

Below is the link to the electronic supplementary material.

Supplementary Material 1

## Data Availability

The datasets generated and/or analysed during the current study are not publicly available due privacy protection and ethical obligations but are available (in deidentified form) from the corresponding author on reasonable request.
