# Supplementary material for: Factors influencing critical thinking in simulation-based maternal-child nursing education among undergraduate nursing students: a mixed methods study
Source: BMC Nurs. 2025 Apr 7;24:389. doi: 10.1186/s12912-025-03016-w (PMC11978189; doi:10.1186/s12912-025-03016-w)
Supplement: Supplementary file 1 — Supplementary Material 1 [file 12912_2025_3016_MOESM1_ESM.pdf]

Focus Group Interview Guide

Introductory Questions

1. When you hear the term "simulation-based learning", what comes to mind? How would you describe it?
2. What are your expectations regarding learning through simulation-based learning? Why do you have these expectations?
3. Have you ever experienced learning through simulation-based learning? If so, how did you feel about it?
4. For those who have had practical learning experiences using simulation-based learning, what do you perceive as the strengths and weaknesses of this approach? Why?

Main Discussion Questions

5. How do you feel about your past clinical learning experiences in maternal-newborn and midwifery practice (antenatal unit, labor and delivery unit, postpartum unit, and complications/infectious disease unit)? Could you share your thoughts?
6. What are your opinions on using simulation-based learning for clinical practice in situations where access to real clinical settings is limited?
7. In your view, what constitutes the success of simulation-based learning in maternal-newborn and midwifery practice? Why?
8. What factors do you believe contribute to the success of simulation-based learning in maternal-newborn and midwifery practice? Why?
9. What challenges or barriers do you think hinder the success of simulation-based learning in maternal-newborn and midwifery practice? Why?
10. What aspects of simulation-based learning in maternal-newborn and midwifery practice did you find most enjoyable? Why?
11. What areas do you think need improvement or further development in simulation-based learning for maternal-newborn and midwifery practice? Why?
12. In your opinion, how has simulation-based learning in maternal-newborn and midwifery practice impacted your knowledge and skills in nursing and midwifery?
13. What factors do you think contribute to developing confidence in your midwifery skills through simulation-based learning (antenatal unit, labor and delivery unit, postpartum unit, and complications/infectious disease unit)? Why?
14. How would you like the practical training in maternal-newborn and midwifery practice to be structured to enhance your confidence in your midwifery competence for the future?

Additional Discussion Questions

15. If you had the opportunity to give advice to junior students preparing to undertake maternal-newborn and midwifery practice, what recommendations would you give them? Why?

Field Note Form

Interview Date: ……………………………………………**Start - End Time:** …………………………………

Learning Unit:

☐ Antenatal Care ☐ Labor and Delivery ☐ Postpartum Care ☐ Complications and Infections

Participant Code:

1. □□□ 2. □□□ 3. □□□ 4. □□□ 5. □□□ 6. □□□

1. Interview Location

……
……
……

2. Characteristics of the Interviewee

……
……
……

3. Continuity of the Interview

……
……
……

4. Reflective Notes

……
……
……

5. Key Concepts Identified from the Interview

……
……
……

6. Challenges and Obstacles

……
……
……

7. Topics to Explore Further in the Next Interview

……
……
……
